# Draft Genome Sequence of the Causative Antigen of Summer-Type Hypersensitivity Pneumonitis, *Trichosporon domesticum* JCM 9580

**DOI:** 10.1128/genomeA.00651-16

**Published:** 2016-07-07

**Authors:** Otomi Cho, Tomoe Ichikawa, Sanae Kurakado, Masako Takashima, Ri-ichiroh Manabe, Moriya Ohkuma, Takashi Sugita

**Affiliations:** a Department of Microbiology, Meiji Pharmaceutical University, Noshio, Kiyose, Tokyo, Japan; b Department of Microbial Science and Host Defense, Meiji Pharmaceutical University, Noshio, Kiyose, Tokyo, Japan; c Japan Collection of Microorganisms, RIKEN BioResource Center, Koyadai, Tsukuba, Ibaraki, Japan; d Division of Genomic Technologies, RIKEN Centre for Life Science Technologies, Suehiro-cho, Tsurumi-ku, Yokohama, Kanagawa, Japan

## Abstract

Here, we report the draft genome sequence of *Trichosporon domesticum* JCM 9580, isolated from the house of a patient with summer-type hypersensitivity pneumonitis (SHP) in Japan. This genomic information will help elucidate the mechanisms of the development of SHP.

## GENOME ANNOUNCEMENT

*Trichosporon* species are basidiomycetous yeast-like fungi that are widely distributed in the environment. In western and southern Japan, these microorganisms are often found in wooden houses during the hot, humid, and rainy season. Repeated inhalation of arthroconidia produced by *Trichosporon* species leads to the development of type III and IV allergies, known as summer-type hypersensitivity pneumonitis (SHP), for which cough and fever are the chief complaints (1). Serologically, *Trichosporon* species are divided into three groups; representative species of the causative agent in each group are *Trichosporon dermatis* for group I, *Trichosporon asahii* for group II, and *Trichosporon domesticum* and *Trichosporon montevideense* for group III (2, 3). *T. domesticum* JCM 9580 was first isolated from the house of an SHP patient in Japan as a novel yeast species (4). The causative antigen from this species is a cell wall polysaccharide containing arabinose (5). Genomic information for this organism should be useful for elucidating the mechanisms of the development of SHP.

Genomic DNA was isolated from the freeze-dried cell mass according to the method of Raeder and Broda (6) and purified using Genomic-tip 100/G (Qiagen KK, Tokyo, Japan), according to the manufacturer’s instructions. Two libraries with approximate insert sizes of 240 and 3 kbp were prepared from DNA using, respectively, a TruSeq DNA PCR-free library preparation kit and Nextera mate-pair sample preparation kit, with some modifications (7). Genome sequencing was accomplished on an Illumina HiSeq 2500 platform for the paired-end library and a MiSeq platform for the mate-pair library, according to the manufacturer’s instructions. The acquired reads were assembled using ALLPATHS-LG (version 52155) (8). Protein-coding genes were predicted using the MAKER annotation pipeline (2.31.8), together with Augustus (3.0.3) and SNAP (2013-02-16), both of which were trained with the *Cryptococcus neoformans* var. *neoformans* JEC21 sequence, and GeneMark-ES (4.21) (9), and annotated using Sma3s (10). The *T. domesticum* JCM 9580 genome comprised 28 scaffolds (93 contigs) with a total size of 26.6 Mbp (204× coverage, 0.1% gaps). Annotation of the genome resulted in a set of 8,170 protein-coding gene models, of which 6,156 and 1,901 were functionally annotated based on the UniProtKB/TrEMBL and UniProtKB/Swiss-Prot databases (release 2015_11), respectively. The average number of exons per gene model was 4.3; the average exon and intron sizes were 345 and 74 bp, respectively, and the G+C content was 58.5%. In addition to protein-coding genes, 375 tRNA genes, 214 genes for small noncoding RNA, and 166 transposons were annotated using tRNAscan-SE (1.23) (11), Infernal cmscan (1.1.1) (12), the Rfam database (release 12.0) (13), RepeatMasker (open-4.0.5) (http://www.repeatmasker.org/), and RepeatRunner (14), based on Repbase (15).

### Nucleotide sequence accession number.

The genomic sequences have been deposited in DDBJ/EMBL/GenBank under accession no. BCFW00000000. The annotations are available via the homepage of the Japan Collection of Microorganisms (JCM) at the RIKEN BioResource Center (http://www.jcm.riken.jp/cgi-bin/nbrp/nbrp_list.cgi).
